# Dynamic Characterization and Damping Enhancement Mechanism of Carbon Fiber Reinforced Hybrid Structures for Aerospace Electronics

**DOI:** 10.3390/polym18040516

**Published:** 2026-02-19

**Authors:** Jun Rao, Qiaoxin Zhang, Yu Feng, Meng Wei, Wentao Yang

**Affiliations:** 1 School of Mechanical and Electronic Engineering, Wuhan University of Technology, Wuhan 430070, China; rao20211214@163.com (J.R.); 15774549727@163.com (Y.F.); 2 Institute of Advanced Material and Manufacturing Technology, Wuhan University of Technology, Wuhan 430070, China; 3 Hubei Anxin Intelligent Technology Co., Ltd., Wuhan 430070, China; weim@hubeianxin.com (M.W.); rlp666888@163.com (W.Y.)

**Keywords:** Carbon Fiber Reinforced Polymer (CFRP), display and control console (DCC), lightweight design, vibration damping, finite element analysis

## Abstract

In modern aerospace cockpits, the display and control console (DCC) serves as a critical human–machine interface. Light weight is particularly important in this industry, especially for key equipment such as the DCC. To address the excessive weight of aluminum alloy DCCs while achieving desirable mechanical properties and vibration-damping performance, this study developed a Carbon Fiber Reinforced Polymer (CFRP) DCC; its superior performance was verified through finite element analysis (FEA) and a vibration test. Compared with conventional aluminum alloy structures, the newly designed DCC achieves approximately a 40% weight reduction while meeting all rigidity, strength, and vibration requirements. This study successfully demonstrates the feasibility of using CFRP to replace aluminum alloy in aircraft DCC and provides a systematic design methodology for similar structures.

## 1. Introduction

Carbon Fiber Reinforced Polymer (CFRP) has become a vital material in modern industries due to its high strength-to-weight ratio, excellent corrosion resistance, and higher inherent damping than many metallic materials. These properties, combined with a specific strength often exceeding that of metals, have led to its widespread use in aerospace and automotive applications [1,2]. In aerospace, CFRP is widely used in primary structures such as fuselages and wings, significantly reducing aircraft weight, improving fuel efficiency, and enhancing structural strength to meet the dual demands of lightweight design and reliability [3,4]. Similarly, in the automotive industry, CFRP is used in body frames, engine components, bumpers, and heat shields, contributing not only to vehicle weight reduction but also to improved safety through enhanced crash energy absorption, thus advancing energy-saving initiatives [5,6]. Other studies have explored CFRP’s potential for enhanced energy efficiency, integrated molding, and sustainable design, contributing to the development of lightweight mechanical equipment frames [7,8,9,10]. Despite these advantages, vibration mitigation and damping enhancement in CFRP structures remain active research topics, because damping performance is highly sensitive to matrix formulation, reinforcement architecture, and structural configuration. For instance, modifying CF/EP systems using micro- or nanoscale inclusions has been reported to improve damping while maintaining mechanical performance, although the overall benefit is strongly dependent on inclusion content and dispersion/processing conditions [11,12]. Beyond particulate approaches, architected carbon-based networks and graphite-filled elastomeric systems have also been shown to increase the loss factor and broaden the effective damping window, illustrating the potential of interfacial and microstructural energy-dissipation mechanisms for vibration control [13,14]. In parallel, CFRP research is increasingly oriented toward multifunctionality—such as impact resistance, acoustic absorption, and thermal management—where multiscale reinforcements, phase design, and interface quality play decisive roles [15,16,17].

The display and control console (DCC) serves as the central human–machine interface in aerospace systems. It performs essential functions such as monitoring equipment status, displaying loads, operational data, and imagery. Conventional DCCs are typically fabricated from bent aluminum alloy sheets, which satisfy basic lightweight requirements. However, under complex operational environments, including sinusoidal, random, and sawtooth wave vibrations, the vibration performance and structural durability of aluminum alloy DCCs are severely challenged. As a result, their suitability for long-term use in harsh service conditions is significantly reduced [18,19]. Notably, damping performance in composite structures is not only governed by constituent materials but also by structural parameters; for example, co-cured damping-layer concepts and joint-/geometry-related design variables have been demonstrated to markedly influence damping responses in composite assemblies, implying that console-level vibration performance should be treated as a coupled material–structure optimization problem rather than a purely material substitution problem.

Despite significant advancements in the industrial use of CFRP, current research continues to show notable limitations. First, existing studies have predominantly focused on large-scale applications and manufacturing technologies for aerospace and automotive components, with limited optimization analysis of specific structures, such as thin-walled composite laminates. The design of thin-walled channels and frame structures lacks specialized optimization methods, with current approaches either overly generalized or restricted to individual components [20]. Second, research on the DCC is still limited. Although previous studies, including Che’s carbon fiber robot console [18], Luo’s integrated vehicle display console [19], and Sotnik’s ergonomic analysis of console layouts [21], have explored console structures, they fail to address the complex service conditions typical of marine DCCs, such as high-strain-rate failure, long-term environmental effects, and dynamic shock resistance. Furthermore, these studies do not provide a systematic design or verification of metal–composite hybrid structures in console applications [22,23,24,25,26]. Third, while traditional aluminum alloy consoles face challenges in balancing lightweight-design goals with seismic resistance, no dedicated framework exists for the design, manufacturing, and performance validation of CFRP-based consoles. In addition, the recent literature suggests that damping enhancement and multifunctional performance improvements can be highly dependent on modifier content/dispersion and interface quality, and that structural-parameter tuning may be equally critical—yet these insights have not been systematically translated into DCC-oriented thin-walled frame/channel design and verification workflows. This gap impedes the technological transition from general CFRP structures to specialized DCC components [27].

To address the research gaps and engineering challenges outlined above, this study presents a novel design scheme and manufacturing process for a CFRP DCC. This approach resolves the conflict between lightweight objectives and shock resistance in traditional metal DCC, while bridging the design gap for specialized CFRP console structures. First, based on the mechanical properties of CFRP prepreg laminae, and while retaining metal fixtures for external displays and keyboards, the remaining metal substructures are replaced with composite materials. Structural parameters are optimized by adjusting the laminate thickness. Next, a comprehensive finite element model of the console is developed to compute structural stiffness and weight, as well as to evaluate the static, dynamic, and modal characteristics of the hybrid metal–composite structure. Finally, by comparing the performance of the proposed design with that of a conventional aluminum alloy console, the optimal composite material specification and manufacturing process are determined, and the effectiveness of the design is validated through experimental testing.

## 2. Materials and Methods

### 2.1. Material

**Figure 1:**
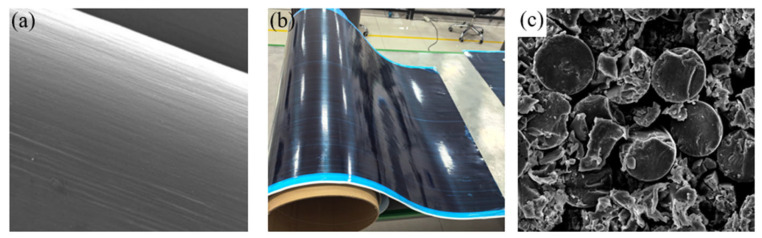
Figures related to CFRP: (**a**) Surface morphology of carbon fiber filaments; (**b**) T300-grade prepreg; (**c**) cross-section of CFRP laminate.

The DCCs investigated in this study are fabricated from aluminum alloy and T300-grade CFRP prepreg. The prepreg is produced by Zhongfu Shenying Carbon Fiber Co., Ltd., with a fiber volume fraction of 57% and a resin volume fraction of 43%. The nominal thickness of a single prepreg ply is 0.2 mm. Figure 1 shows figures related to CFRP, and the detailed material parameters are listed in Table 1.

### 2.2. Structural Design

**Figure 2:**
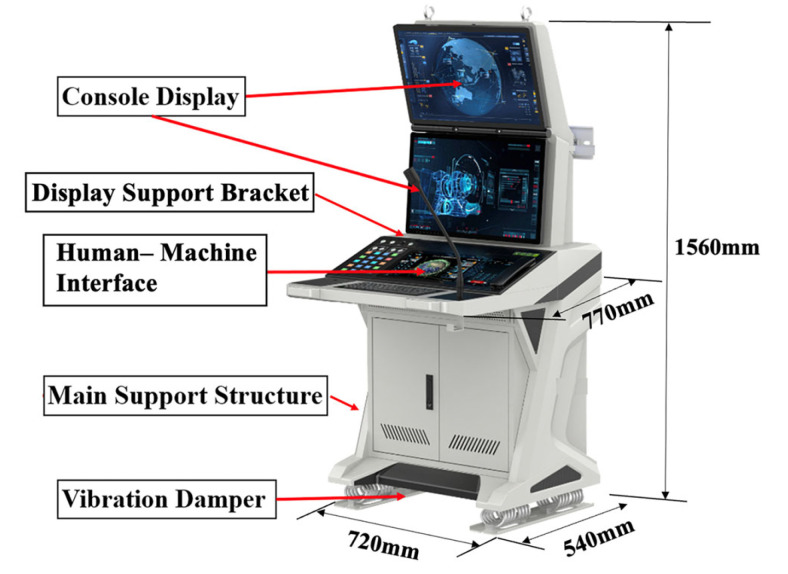
Main dimensions and components of aluminum alloy DCC.

The baseline DCC, constructed from aluminum alloy, serves as the control group and is depicted in Figure 2 along with its principal dimensions. This conventional assembly possesses a total mass of 133 kg, of which the primary structural skeleton comprises 69.5 kg. To achieve weight reduction while maintaining dynamic stability, the implementation of a CFRP substitution strategy was proposed.

Unlike the monolithic metal design, the CFRP DCC utilizes a modular assembly architecture. This approach divides the console into discrete components—specifically, the main frame, display support, and console panel—to optimize fiber continuity and manufacturability. The structural components are designed with a wall thickness of 5 mm. A quasi-isotropic layup sequence is employed to balance mechanical properties, featuring a ply ratio of 25% (0°), 25% (90°), and 50% (±45°). The specific laminate schedule consists of an outer 3 K twill weave for surface quality, followed by alternating unidirectional prepreg layers [0/45/90/−45] s. To prevent resin-rich areas and fiber bridging in curved sections, rolled unidirectional fabric rods are utilized as fillers. The optimized CFRP DCC achieves a structural mass of approximately 41.5 kg, representing a 40% mass reduction compared to the aluminum baseline. Aluminum 6061 alloy is retained only for high-precision interfaces, such as the display connectors and inclined mounting islands.

### 2.3. Fabrication

**Figure 3:**
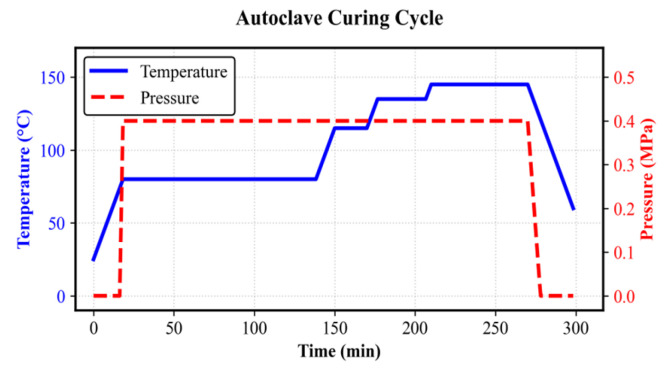
Autoclave curing cycle of CFRP DCC.

The CFRP components were manufactured using an autoclave molding process, selected to ensure high consolidation and minimize void content. Compared to compression molding or RTM, autoclave curing provides superior interlaminar shear strength and dimensional stability for complex geometries. The curing process followed a multistage temperature and pressure ramp, as shown in Figure 3. A consolidation pressure of 0.4 MPa was applied at 80 °C and maintained for 300 min. The thermal cycle included varying dwell times at 80 °C, 115 °C, 135 °C, and 145 °C to ensure complete crosslinking, followed by a controlled cooling rate of 3 °C/min to mitigate residual thermal stresses [28,29].

**Figure 4:**
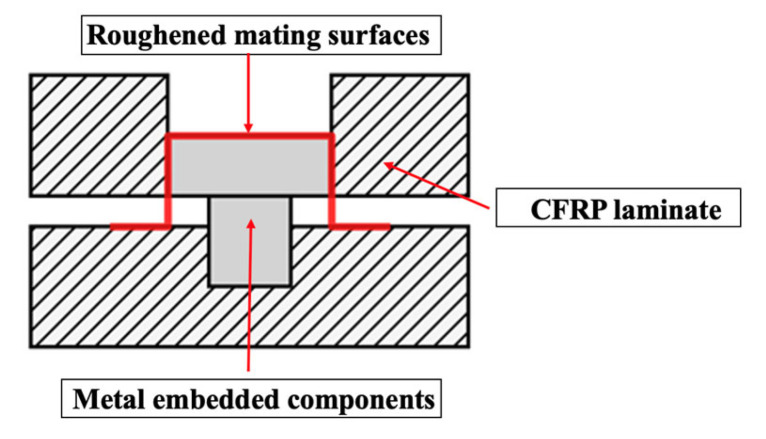
Local reinforcement mechanism diagram.

To ensure robust mechanical connections, metal inserts were integrated using co-bonding techniques, as illustrated in Figure 4. For high-load points, inserts were sandblasted, wrapped in unidirectional fabric, and co-cured with the laminate. For secondary attachment points, inserts were bonded using structural adhesive applied to roughened mating surfaces and mechanically secured during the cure.

### 2.4. Methodology

CFRP prepreg consists of carbon fibers embedded in a resin matrix, with the resin conferring significantly higher damping capacity to the laminate than aluminum alloys. In the DCC, which comprises multiple components, the additive damping contributions from each part yield superior overall vibration damping performance. The viscoelastic nature of the epoxy resin in CFRP effectively dissipates vibrational energy, thereby enhancing the structural dynamic response. Consequently, CFRP-based DCCs outperform aluminum-based counterparts in mechanical performance under equivalent vibration conditions.

The following section presents a theoretical analysis of damping in composite laminates. Based on the energy dissipation principle, the damping capacity of a structure is defined as the ratio of dissipated energy to maximum strain energy per vibration cycle [30,31].

(1) $$D=2\pi \eta =\frac{\Delta U}{U}$$

where D is the damping ratio, η is the damping loss factor, and ΔU and U represent the dissipated energy and the total strain energy stored in one vibration period. The damping loss factor of the structure can be expressed as:

(2) $$\eta =\frac{\sum_{k=1}^{n}\eta_{ij}U_{ij}^{k}}{\sum_{k=1}^{n}U_{ij}^{k}}$$

where $U_{ij}^{k}$ is the sum of the strain energy of the kth cell of the structure generated by the stress $\sigma_{ij}$ of the layer, $\eta_{ij}$ is the damping loss factor in the corresponding direction, 1 refers to the positive axis direction, 2 refers to the direction perpendicular to the fiber, and 3 refers to the thickness direction. Under the small deformation assumption and the linear elasticity assumption, the strain energy generated by each unit can be calculated by Equation (3):

(3) $$U_{ij}^{k}=0.5\int \sigma_{ij}^{k}\varepsilon_{ij}^{k}dV^{k}(i,j=1,2,3)$$

$\sigma_{ij}^{k},$ $\varepsilon_{ij}^{k}(i,j=1,2,3)$ represent the stress and strain components in the kth unit of the laminate, respectively. $V^{k}$ is the integral volume of unit k.

The CFRP DCC is composed of many parts; the proportion of strain energy loss varies between the different parts, so the definition of strain energy loss in different directions of each part of the structure is proposed as follows:

(4) $$SE_{ij}^{p}=\;100\times \frac{∆U_{ij}^{p}}{max\left\{∆U_{total}^{1},∆U_{total}^{2}\ldots \ldots ,∆U_{total}^{n}\right\}}$$

$U_{\mathrm{total}}^{n}$ represents the sum of strain energy loss, $∆U_{ij}^{p}$ is the strain energy loss generated by the stress $\sigma_{ij}$ in the part p. $SE_{ij}^{p}$ represent the proportion of strain energy loss generated by stress $\sigma_{ij}$ in the part p of the structure. The component with the largest strain energy loss in the structure is:

(5) $$SE^{p_{max}}=\;\sum_{i=1}^{3}\sum_{j=1}^{3}SE_{ij}^{p}=100\;(i,j=1,2,3)$$

For the whole DCC structure, during one vibration period, total dissipated energy and total strain energy can be expressed as:

(6) $$∆U=\sum_{k=1}^{n}\sum_{i=1}^{3}\sum_{j=1}^{3}2\pi \eta_{ij}U_{ij}^{k}2\pi \sum_{k=1}^{n}\sum_{i=1}^{3}\sum_{j=1}^{3}\frac{1}{2}\int \eta_{ij}\sigma_{ij}^{k}\varepsilon_{ij}^{k}dV^{k}$$

(7) $$U=\sum_{k=1}^{n}\sum_{i=1}^{3}\sum_{j=1}^{3}U_{ij}^{k}=\sum_{k=1}^{n}\sum_{i=1}^{3}\sum_{j=1}^{3}\frac{1}{2}\int \sigma_{ij}^{k}\varepsilon_{ij}^{k}dV^{k}(i,j=1,2,3)$$

The damping loss factor of the structure is:

(8) $$\eta =\frac{\Delta U}{2\pi U_{max}}=\frac{2\pi \sum_{k=1}^{n}\sum_{i=1}^{3}\sum_{j=1}^{3}\frac{1}{2}\int \eta_{ij}\sigma_{ij}^{k}\varepsilon_{ij}^{k}dV^{k}}{2\pi \sum_{k=1}^{n}\sum_{i=1}^{3}\sum_{j=1}^{3}\frac{1}{2}\int \sigma_{ij}^{k}\varepsilon_{ij}^{k}dV^{k}}=\;\frac{\sum_{k=1}^{n}\sum_{i=1}^{3}\sum_{j=1}^{3}\int \eta_{ij}\sigma_{ij}^{k}\varepsilon_{ij}^{k}dV^{k}}{\sum_{k=1}^{n}\sum_{i=1}^{3}\sum_{j=1}^{3}\int \sigma_{ij}^{k}\varepsilon_{ij}^{k}dV^{k}}$$

There are six damping loss factors in 6 directions of the composite material, continuous fiber composites have transverse isotropy, and the plane perpendicular to the fiber direction is isotropic; therefore, $\eta_{12}=\eta_{13}$, $\eta_{22}=\eta_{33}$ are obtained. The contribution of the damping loss factor in the 2–3 direction of the laminate structure to the actual damping of the whole structure is small; thus, the influence of $\eta_{23}$ can be ignored [32].

The damping loss factor of the structure is converted into a damping ratio:

(9) $$\zeta =\;\frac{\eta }{\sqrt{4+\eta^{2}}}$$

where ζ represents the structural damping ratio.

For laminated plate structures, a marine display console uses a CFRP frame instead of aluminum alloy, achieving a 13% weight reduction and passing high-intensity impact and random-vibration tests. Vehicle-mounted tactical displays and lightweight display units utilize the high specific strength of CFRP, reducing deformation by 90.7% under extreme impact conditions [33,34].

## 3. Finite Element Analysis

In this study, a geometrically nonlinear finite element analysis was performed for the DCC. Modal simulation is utilized not only to determine natural frequencies for avoiding environmental resonance but also to identify structural weak points through mode shape analysis. Furthermore, random-vibration simulation evaluates the fatigue accumulation and stress levels of the structure throughout its full life cycle within the frequency domain.

**Figure 5:**
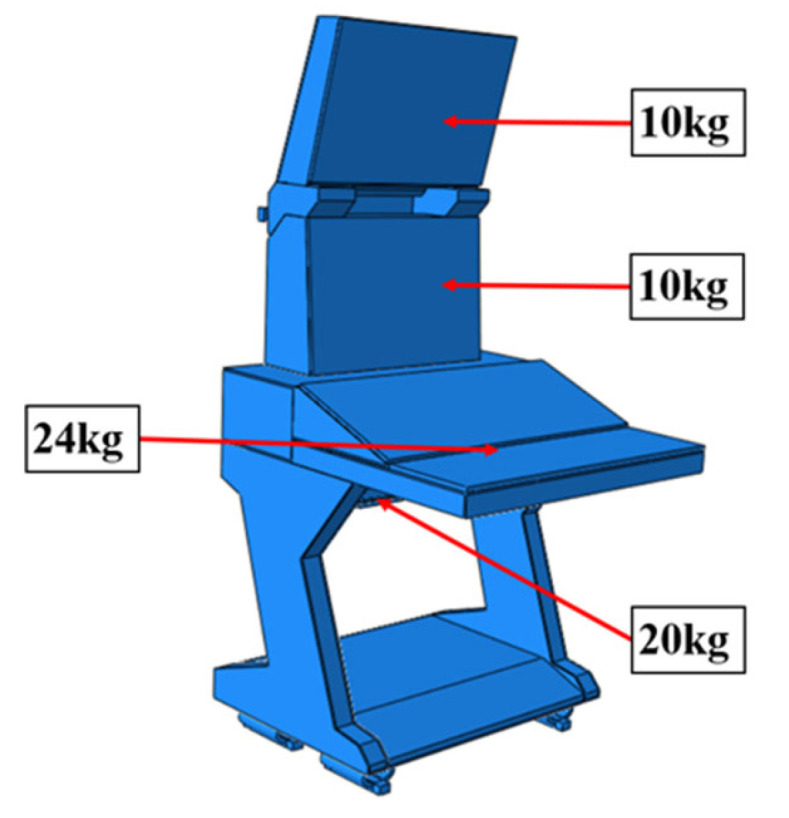
Finite element analysis modal of the DCC.

Solid and shell elements were used to model and assemble the various components made of different materials. Considering the forming characteristics of CFRP laminates, a simplified modeling strategy was adopted for certain regions that are not conducive to efficient numerical computation. These simplifications are consistent with the actual forming process in production, thereby reducing discrepancies between the simulated model and the manufactured structure. All simulations were conducted using the commercial finite element software ABAQUS^®^ 2025 (Dassault Systèmes Simulia Corp., Providence, RI, USA). The finite element model of the DCC is shown in Figure 5, where the distribution of counterweights in different regions is also indicated. For the finite element modeling of the entire display console, the continuous shell in finite element analysis is used to model the layer of prepreg, in order to achieve consistency with the actual manufacturing process. More specifically, the continuous shell C3D8R element is used to set the performance, and the swept mesh analysis is employed to simulate the actual situation more reasonably. When setting the parameters related to damping characteristics, the relevant parameters at room temperature are used.

For the CFRP DCC, the shock absorbers and hinges retain their original metallic materials, while the remaining parts are made from T300 prepreg and modeled using ABAQUS^®^ C3D8R elements. The laminate stacking sequence is [0/45/90/−45/] s, and the corresponding material properties are listed in Table 2. The counterweight of the DCC mainly consists of the display and other electrical equipment, which must be fully included in the simulation to obtain accurate results. To accurately represent the mass distribution without artificially stiffening the structure, MPCs (Multi-Point Constraints) were utilized to distribute the weight of the electronic payloads onto the structural hard points. This approach ensures that the inertial effects and energy distribution under actual operating conditions are correctly simulated.

Modal analyses are then performed for both types of display consoles to compare their natural frequencies. In addition, random response analyses under several typical operating conditions are carried out to evaluate their mechanical performance. In the finite element model, certain geometric features that are computationally expensive to model explicitly in the simulation, such as chamfers and bolt holes, are simplified. These simplifications do not significantly affect the overall accuracy of the simulation results.

### 3.1. Modal Analysis

Modal analyses of the two DCCs were carried out using ABAQUS. The DCC is subjected to a variety of vibration environments, its natural frequencies are critical parameters governing dynamic performance. Appropriately designed natural frequencies help avoid resonance, which can otherwise lead to excessive vibration and potential structural damage. Compared with metallic materials, CFRP exhibits superior damping properties, allowing it to absorb vibration energy and thereby reduce structural damage and radiated noise. Fixed boundary conditions were applied at the vibration damper locations to compute the constrained modes. The first five natural frequencies and corresponding mode shapes of the DCCs were obtained from the finite element analysis, as summarized in Table 2.

The modal finite element analysis of the DCC made from two different materials shows that, for the first five modes, the natural frequencies of the CFRP DCC are higher than those of the aluminum alloy configuration. However, the maximum deformation of the CFRP DCC at the corresponding frequencies is smaller. These results indicate that the use of CFRP provides clear advantages for the design and manufacture of DCC.

Discrepancies between simulated modal frequencies and experimental values primarily stem from non-ideal contact at connection interfaces and the assumption of uniform material damping. The simulation treats all connections as perfectly coupled, thereby neglecting the local stiffness hardening induced by assembly preload, as well as the nonlinear, asymmetric stiffness variations caused by potential looseness. Consequently, such idealizations typically result in the simulated natural frequencies being slightly higher than the experimentally measured values.

### 3.2. Evaluation of Random-Vibration Results Based on RMISES

The evaluation of the random-vibration analysis is based on the contour of the RMISES (root mean square von Mises stress). It is important to note that the RMISES value output by ABAQUS represents a statistical quantity corresponding to the 1σ (one-sigma) stress level. For a Gaussian random process, this means that the instantaneous von Mises stress is expected to remain below the RMISES value for approximately 68.27% of the time. To ensure a high level of structural reliability, a more conservative stress measure is adopted. The 3σ stress, obtained by multiplying the RMISES value by three, is used as the primary evaluation index. This 3σ level corresponds to a 99.73% probability that the stress will not exceed this value. Accordingly, the fundamental structural safety criterion is defined as:

3σ Stress = 3 × RMISES < σy

where σy is the material yield strength.

In practice, the post-processing procedure involves extracting the maximum RMISES value from the final cumulative analysis step. The corresponding 3σ stress is then calculated and compared with the allowable stress, determined by the yield strength or fatigue strength, as appropriate. Satisfaction of this criterion confirms that the structure is adequate under the specified random-vibration environment.

For the matrix phase of CFRP, the use of von Mises stress provides a reasonable approximation of the shear strain energy distribution, thereby assisting in the localization of stress concentration zones. Despite the inherent anisotropy of the composite, the von Mises stress contour intuitively highlights critical regions within the matrix phase, facilitating the identification of potential failure initiation sites

#### 3.2.1. Transportation Vibration Simulation

**Figure 6:**
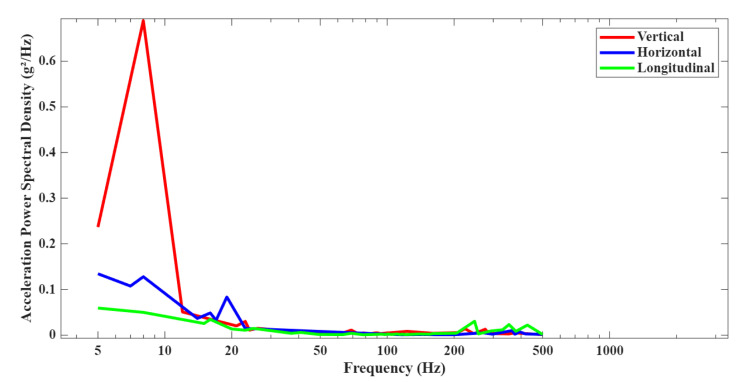
The vibration environment of the combined wheeled vehicle.

During transportation, the DCC may be subjected to a variety of vibration environments. In this study, the vibration loading is defined according to the combined wheeled-vehicle random-vibration profile specified in Figure C3 of GJB150.16A. The corresponding vibration spectrum covers a frequency range from 5 Hz to 500 Hz. The acceleration power spectral density (PSD) curves in the three orthogonal directions are shown in Figure 6, and the corresponding corner (break) frequencies are listed in Table 3.

**Figure 7:**
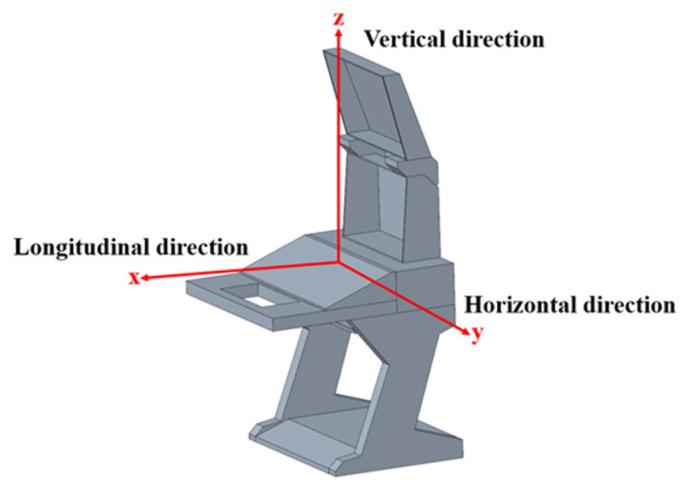
Loading directions.

Vibrations are applied along the x (Longitudinal), y (Horizontal), and z (Vertical) directions of the equipment coordinate system, as shown in Figure 7. These loading conditions are input into the software to perform a simulation analysis of the DCC.

**Figure 8:**
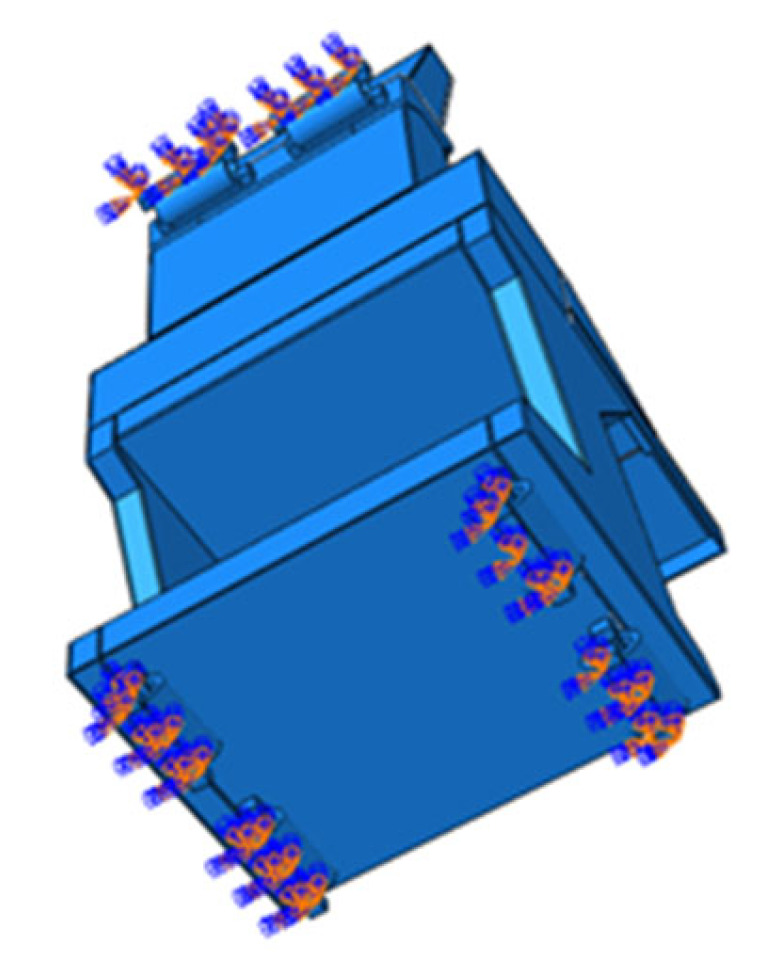
Boundary conditions.

Random response analyses of the DCC were performed separately along the X, Y, and Z directions. The PSD curves given in Table 3 were defined in ABAQUS, with fixed constraints applied to the six shock absorbers, as shown in Figure 8.

The root mean square von Mises stress (RMISES) was extracted to assess the average stress response of the console. In combination with the modal analysis results, peaks in the response at the natural frequencies were identified to determine whether resonance was excited. Structural failure of the laminate was evaluated using the 3σ failure criterion. The simulation results for the CFRP DCC were then compared with those of the aluminum alloy console, and all outcomes are summarized in Table 3.

From the random-vibration simulations of the two DCC configurations under three-directional loading, it can be seen that the differences in maximum displacement and maximum stress are small. This indicates that both consoles exhibit satisfactory dynamic performance. In the evaluation of the random-vibration results in ABAQUS, the primary quantity of interest is the RMISES (root mean square von Mises stress), which corresponds to the 1σ stress level. Statistically, this implies a 68.27% probability that the instantaneous stress remains below this value. Following common engineering practice, the 3σ stress level, calculated as 3 × RMISES, is adopted as the assessment criterion, corresponding to a 99.73% probability that the stress does not exceed this level. Structural integrity is considered acceptable if the 3σ stress is lower than the material yield strength. This provides a conservative and reliable basis for evaluating structural performance under random-vibration loading. For the CFRP DCC, the calculated 3σ stress level is significantly lower than the yield strength of the material, indicating a sufficient safety margin. Therefore, under the considered random-vibration conditions, the CFRP DCC can operate safely and reliably.

#### 3.2.2. Bump Shock

The DCC must satisfy the bump shock requirements specified in GJB150.16A-2009. Under this operating condition, the power spectral density is constant acceleration, with a starting frequency of 1 Hz and a cutoff frequency of 100 Hz. The power spectral density value within this frequency range is 0.001 g ^2^/Hz. This PSD is mainly applied in the vertical direction (z direction). The vibrational FEA result under this condition is shown in Table 4.

Similarly, under the RMISES-based evaluation, the 3σ stress of the CFRP DCC remains below the allowable limit. This result indicates that the CFRP DCC can safely withstand this operating condition.

#### 3.2.3. Terminal Peak Sawtooth Shock

**Figure 9:**
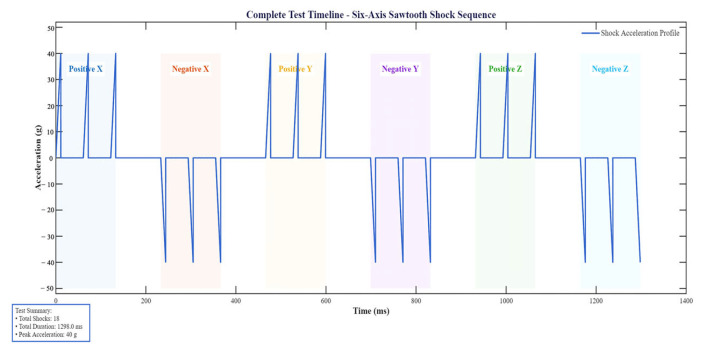
Six-axis terminal peak sawtooth wave loading.

According to the functional impact test procedure specified in GJB150.18A-2009, the test is carried out as follows. First, an initial inspection is performed to establish the baseline performance of the test item. The item is then mounted on the impact platform in a non-operational state. A post-peak sawtooth shock pulse with a minimum peak acceleration of 40 g, which is representative of aerospace environments, and a pulse duration of 11 ms is applied. The impacts are applied along six orthogonal directions: ±x, ±y, and ±z, with three shocks in each direction, as shown in Figure 9. After all impact tests are completed, the test item is returned to its normal configuration, and a final inspection is performed to assess any changes in performance.

According to the GJB150.18A-2009, corresponding boundary conditions and load inputs are applied in ABAQUS. Three directions of acceleration sawtooth wave inputs are applied, and the simulation results are shown in Table 5.

According to the simulation results in the table, the CFRP DCC exhibits better performance than the aluminum alloy console under sawtooth wave loading, with lower maximum deformation and lower maximum stress. This demonstrates that the CFRP console has superior mechanical characteristics. In addition, the Tsai–Wu failure index for the carbon fiber composite remains well below 1, confirming its structural reliability and safety under the specified operating conditions.

## 4. Experiments

**Figure 10:**
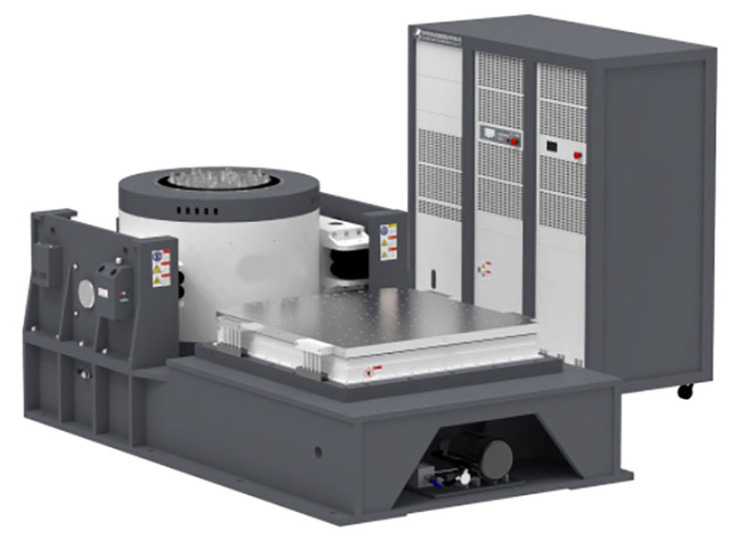
Electric vibration testing system.

A random-vibration test was carried out on the DCC using an STI electrodynamic shaker (DC-10000-100) with a rated force of 98 kN; the device is shown in Figure 10. The shaker provides a maximum displacement of 51 mm (peak), a maximum velocity of 5 m/s and a maximum acceleration of 50 g within a usable frequency range of 1–2500 Hz. The test in the present study was performed in the vertical direction. In actual service conditions, due to the console’s center of gravity height and mounting configuration, the vertical direction typically represents the dimension with the lowest stiffness and highest energy transfer efficiency.

The DCC assembly was mounted on the shaker slip table through a dedicated fixture using the same interface points as in simulation. The total fixture and test article mass was within the capacity of the shaker and power amplifier. The power amplifier driving the shaker had a maximum input voltage of 9 V, while the controller drive output for this test was limited to 5 V to protect the system.

### 4.1. Instrumentation and Control

Random vibration was controlled by a multi-channel vibration controller. The input configuration is summarized in Table 6. Charge-type accelerometers were used on the control channels, with typical sensitivities of 27.32–28.32 pC/g and 10.32 pC/g, and AC single-ended accelerometer channels (100 mV/g) were available for additional monitoring. One channel was used as the control channel mounted close to the shaker reference point, and several channels were reserved for monitoring the response of the console at critical locations.

The main control parameters were as follows: maximum analysis frequency—2000 Hz, 1600 spectral lines, frequency resolution—1.25 Hz, frame time—0.8 s, linear averaging over 8 frames and weighted averaging over 40 frames. The permitted level change rate was 20 dB/s with a level step of 10%. Sigma-clipping was disabled, and resumption from abort was not allowed, in order to ensure conservative test conditions.

### 4.2. Target Spectrum and Test Procedure

The random-vibration input followed a broadband acceleration power spectral density (PSD) profile between 5 and 500 Hz. The overall target root mean square (RMS) acceleration of the spectrum was 2.21 g, corresponding to an RMS velocity of 0.343 m/s and an RMS displacement of 8.4 mm. The associated peak values were 6.62 g, 1.03 m/s and 50.4 mm peak-to-peak, respectively. The capability of the shaker (50 g, 5 m/s, 102 mm peak-to-peak) exceeded these demands with sufficient margin. The PSD was defined by 26 breakpoints from 5 to 500 Hz with specified left and right spectral slopes in dB/octave, as summarized in Table 3. This definition results in a spectrum whose main energy is concentrated in the low-frequency band (5–90 Hz), with additional contributions in the 120–300 Hz range, representative of the transportation environment of the console.

To protect the test article, alarm and abort limits were set relative to the overall RMS acceleration: the alarm band was [1.56, 3.12] g and the abort band was [1.11, 4.40] g, bracketing the 2.21 g target level. These limits were applied to the control channel during the entire test. The test was executed following a stepped-level schedule (Table 1): the PSD level was first ramped to 30%, 50% and 80% of the target level, each held for 10 s for verification, and then raised to 100% and maintained for 1 h at full level. This procedure allowed the integrity of the console and fixture to be checked before the full-severity exposure.

### 4.3. Test Results

**Figure 11:**
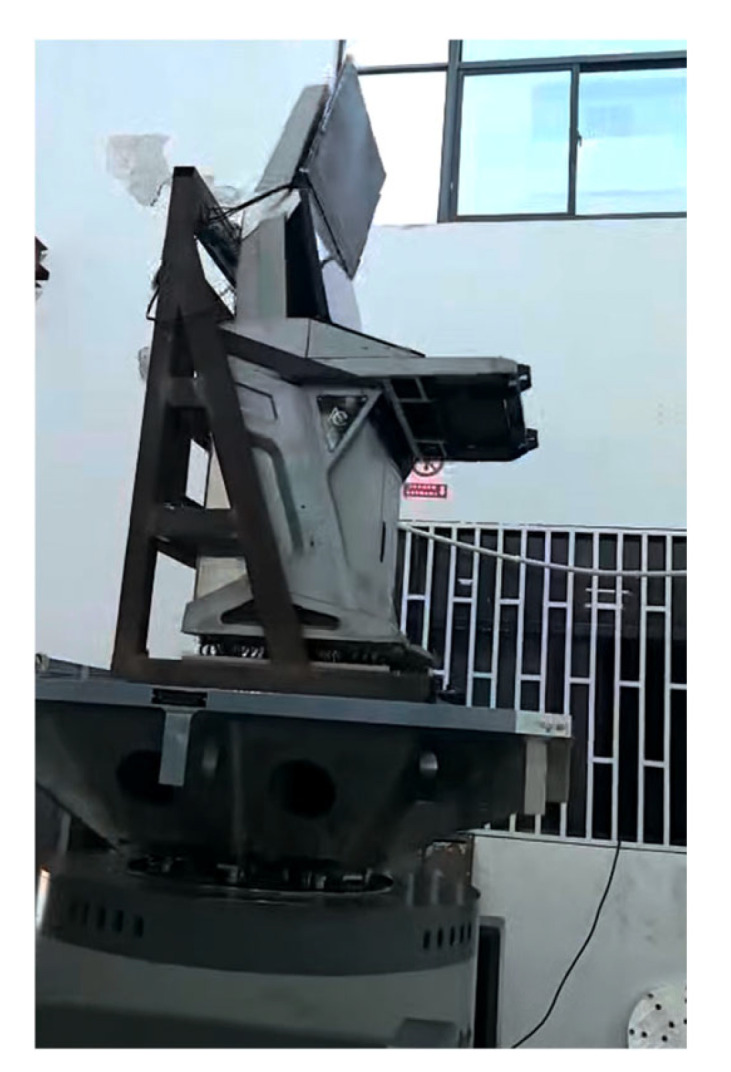
The DCC on the vibration device.

During the 1 h dwell at the 100% test level, the controller maintained the effective RMS acceleration at 2.27 g, within 3% of the target. No system aborts or spectral line exceedances beyond the abort limits occurred. Figure 11 shows the DCC on the testing equipment.

**Figure 12:**
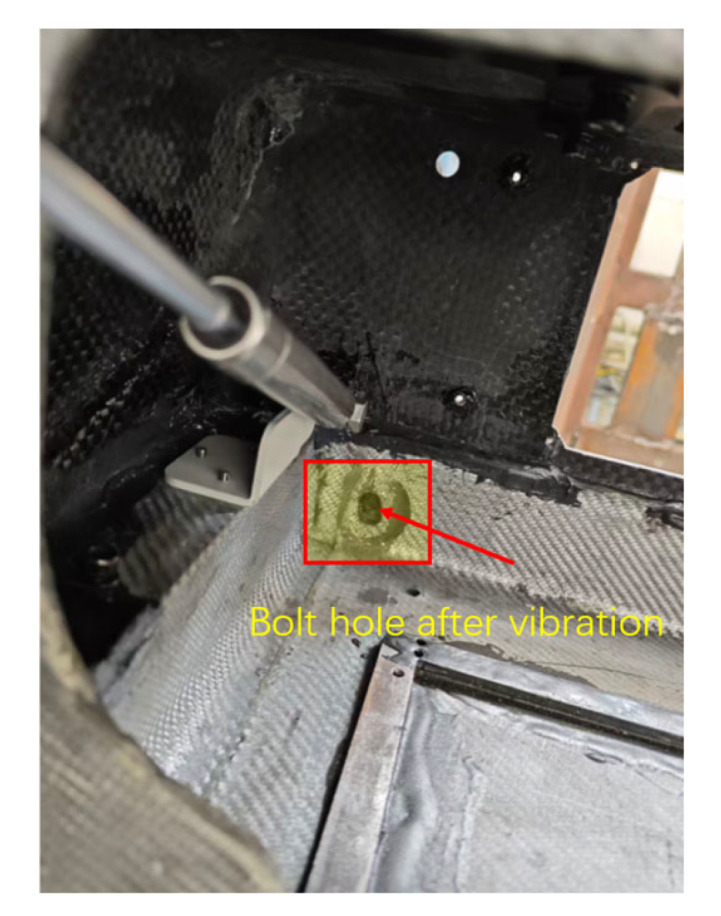
Stress concentration leads to local expansion of embedded bolt holes.

However, post-test visual inspection revealed localized damage. Specifically, minor hole elongation was observed at the bolted joints connecting the display bracket to the CFRP frame, as shown in Figure 12. While the global dynamic response remained stable, which indicated sufficient structural rigidity, these local failures suggest significant stress concentrations at the metal–composite interface. This phenomenon was not fully captured by the simplified MPC/Coupling constraints in the FEA model.

### 4.4. Discussion

(1) The bolt-hole elongation observed in the experiments represents a typical bearing failure mechanism in composite materials subjected to alternating stresses.

In the finite element analysis, the initial model utilized Multi-Point Constraints (MPCs) to simplify the connections, which inadvertently underestimated the local stress gradients. For CFRP laminates under unidirectional tension, the hole edge is significantly influenced by the layup orientation. Under the high-cycle fatigue loading induced by random vibration, the matrix surrounding the hole experiences plastic flow or micro-crack propagation. This degradation reduces the effective contact area between the bolt shank and the hole wall, ultimately resulting in permanent hole expansion. Current optimization strategies recommend the implementation of an interference fit. This technique leverages a pre-compressive residual stress field to counteract a portion of the tensile stresses generated by vibration, thereby enhancing the fatigue life of the joint.

(2) The coupled influence of temperature and humidity, known as hygrothermal effects, constitutes a critical service risk that cannot be overlooked.

Moisture absorption induces plasticization and swelling of the epoxy matrix, which depresses its glass transition temperature. Macroscopically, this manifests as a degradation in the transverse modulus of the composite: water molecules penetrate the fiber/matrix interface, compromising the interfacial shear strength. This weakening of the interface results in enhanced energy dissipation, thereby increasing the structural damping. When reaching moisture saturation, the loss factor of composite structures can increase; however, the fundamental frequency may also concurrently decrease. In the future, more detailed research should be conducted on the impact of this on the display console.

## 5. Conclusions

This study establishes a comprehensive design framework for CFRP DCC tailored to complex aerospace operating conditions. The proposed approach achieves a coordinated improvement in multiple performance metrics, including a 40% reduction in weight, enhanced structural stiffness, and improved vibration resistance. By introducing a hybrid metal–composite architecture, the design maintains structural integrity while ensuring functional practicality. This paper presents a comparative FEA of two DCCs fabricated from distinct materials. The CFRP configuration demonstrates outstanding performance under both explicit dynamic and random-vibration loading conditions, and maintains a fundamental frequency that effectively avoids interference with operational excitation spectra, thereby eliminating resonance risks.

Both theoretical calculations and numerical simulations substantiate the practical feasibility and engineering value of implementing CFRP in console applications. The employed modeling approach significantly reduces development cycles and experimental costs, establishing a reliable reference framework for subsequent physical prototype development.

After conducting vibration tests on the CFRP DCC and benchmarking the results against FEA, no appreciable damage was observed. This experimental outcome is consistent with the simulation predictions, which indicate no significant discrepancy between the measured response and the numerical analysis. It should be acknowledged that the simulated boundary conditions represent idealized scenarios that may diverge from actual operational environments.

This work provides a referenced technical pathway for developing specialized CFRP components in aerospace and related fields, effectively addressing core engineering challenges in balancing lightweight objectives with structural reliability.

## Figures and Tables

**Table 1:** Engineering constants of CFRP prepreg and aluminum alloy.

Notation	Unit	Aluminum Alloy 6061	Carbon/Epoxy Composite (T300/YPH-308)
Young’s modulus (MPa)	E11	71,000	125,000
	E22		8800
	E33		8800
Shear modulus (MPa)	G12	27,000	4510
	G23		3080
	G13		4510
Poisson’s ratio	N12	0.33	0.31
	N23		0.43
	N13		0.31
Mass density (kg/m^3^)	ρ	2700	1600
Ply thickness (mm)	t		0.2

**Table 2:** FEA modal results of natural frequency.

Symbol	Aluminum DCC	CFRP DCC
1st-order/Hz	15	131
Maximum deformation/mm	96.5	24.5
Modal shape	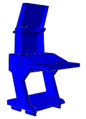	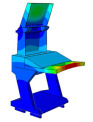
2nd-order/Hz	18.4	136
Maximum deformation/mm	15.8	45.7
Modal shape	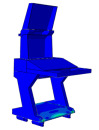	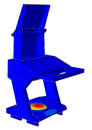
3rd-order/Hz	25	144
Maximum deformation/mm	26.2	16.5
Modal shape	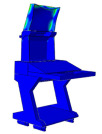	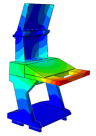
4th-order/Hz	30.2	154
Maximum deformation/mm	74.5	25.7
Modal shape	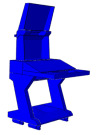	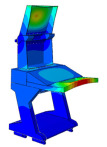
5th-order/Hz	30.3	175
Maximum deformation/mm	58.7	48.5
Modal shape	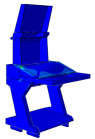	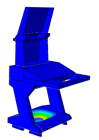

**Table 3:** Transportation vibration simulation results of the two DCCs.

Operating Condition	Tape	U/mm	Mises	RMISES
Vertical direction	CFRP DCC	1.98 × 10^−9^	1.875 × 10^−4^	33.7
	Al DCC	3.436 × 10^−10^	4.12 × 10^−9^	20.2
Horizontal direction	CFRP DCC	7.411 × 10^−10^	2.63 × 10^−4^	15.1
	Al DCC	3.21 × 10^−12^	4.04 × 10^−13^	4.24
Longitudinal direction	CFRP DCC	7.62 × 10^−9^	6.99 × 10^−4^	17.4
	Al DCC	8.42 × 10^−9^	1.692 × 10^−9^	1.86

**Table 4:** Bump shock simulation results of the two DCCs.

Materials	U	S	RMISES	Tsai–Wu
CFRP	4.02 × 10^−4^	3.17	8.65	
Al	2.54 × 10^−7^	2.71 × 10^−6^	3.67	——

**Table 5:** Simulation result of six-axis terminal peak sawtooth wave loading.

Loading Direction	Tape	U/mm	Mises/MPa	Tsai–Wu
X	CFRP DCC	0.23	81.3	7.49 × 10^−2^
	Al DCC	7.9	1400	
Y	CFRP DCC	0.24	98.4	7.73 × 10^−2^
	Al DCC	0.24	92.76	
Z	CFRP DCC	0.22	1356	
	Al DCC	0.25	1407	8.61 × 10^−2^

**Table 6:** Input channel parameter configuration.

Input Channel	Type	Range (V)	Weighting Coefficient	Coupling Method	Sensor Type	Sensitivity
1	Control	10	1	Charge	Acceleration	27.32 pC/(g)
2	Forbidden	10	0	Charge	Acceleration	28.32 pC/(g)
3	Control	10	1	Charge	Acceleration	10.32 pC/(g)
4	Monitor	10	0	Charge	Acceleration	28.3 pC/(g)
5	Forbidden	10	0	AC	Acceleration	100 mV/(g)
6	Control	10	1	Charge	Acceleration	28.2 pC/(g)
7	Forbidden	10	0	AC	Acceleration	100 mV/(g)
8	Forbidden	10	0	AC	Acceleration	100 mV/(g)

## Data Availability

The data presented in this study are available on request from the corresponding author.

## Abbreviations

The following abbreviations are used in this manuscript:

CFRP	Carbon Fiber-Reinforced Polymer
DCC	Display and Control Console
FEA	Finite Element Analysis
